# Evaluation of clinical features and treatment modality of pediatric patients with Steven Johnson syndrome/toxic epidermal necrolysis: a single-center experience

**DOI:** 10.55730/1300-0144.5990

**Published:** 2025-02-05

**Authors:** Funda AYTEKİN GÜVENİR, Vildan Selin ÇAYHAN, Selman Kürşat BALCI, Ragıp DERE, Hatice Irmak ÇELİK, Serhat EMEKSİZ, Ahmet SELMANOĞLU, Zeynep ŞENGÜL EMEKSİZ, Emrah ŞENEL, Emine DİBEK MISIRLIOĞLU

**Affiliations:** 1 Division of Pediatric Allergy and Immunology, Department of Pediatrics, Ankara Bilkent City Hospital, Ankara, Turkiye; 2 Department of Pediatric Surgery, Ankara Bilkent City Hospital, Ankara, Turkiye; 3 Division of Pediatric Intensive Care, Department of Pediatrics, Ankara Bilkent City Hospital, Ankara, Turkiye

**Keywords:** Steven Johnson syndrome, toxic epidermal necrolysis, drug hypersensitivity

## Abstract

**Background/aim:**

Steven Johnson Syndrome (SJS) and Toxic Epidermal Necrolysis (TEN) are rare but life-threatening severe cutaneous drug hypersensitivity reactions. The aim of our study was to evaluate the clinical features of pediatric patients diagnosed with SJS, TEN or SJS/TEN overlap and to examine treatment modalities.

**Materials and methods:**

Patients aged 0–18 years who were followed up with SJS, TEN or SJS/TEN overlap at Ankara Bilkent City Hospital between August 2019 and January 2024 were retrospectively analyzed.

**Results:**

Twelve patients who met the inclusion criteria were included in the study. Five of the patients had SJS, four had SJS/TEN overlap, and three had TEN. Eight of the patients were female, and the median age at presentation was 10.5 (IQR:6–16) years. Ten of the patients had a history of drug use. Eight patients had antibiotics, two had proton pump inhibitors, two had allopurinol, and two had antiepileptic (lamotrigine and valproic acid) use. All 12 patients received IVIG and systemic steroid therapy. Three TEN and four SJS/TEN overlap patients received cyclosporine. Two TEN patients underwent plasmapheresis. The most common long-term sequelae were dermatological sequelae.

**Conclusion:**

SJS/TEN patients should be monitored with a multidisciplinary approach, and if necessary, in the burn intensive care unit. Primary treatment is supportive care. Early initiation of cyclosporine may have a positive effect on the prognosis in patients with SJS/TEN overlap and TEN.

## 1. Introduction

Steven Johnson Syndrome (SJS) and toxic epidermal necrolysis (TEN) are rare, severe cutaneous drug hypersensitivity reactions associated with high mortality and morbidity [1]. Epidermal detachment, keratinocyte necrosis, and blister formation are characteristic [2]. The distinction between SJS and TEN relates to the percentage of body surface area experiencing epidermal detachment. In SJS, body surface area involvement is 10% or less, whereas in TEN it is 30% or more. If it is between 10 and 30%, the condition is known as “SJS-TEN overlap syndrome” [3].

Most but not all cases of SJS/TEN are drug-related (72%–90%) [4]. Mycoplasma infections, viral infections, or bacterial infections may also be responsible[5].

The main treatment involves discontinuing the responsible drug and providing supportive care [6]. Studies have shown the effectiveness of various other treatment regimens as well, including intravenous immune globulin (IVIG), systemic steroids and cyclosporine [7]. However, studies, particularly those involving children, are generally limited to case series.

Steven Johnson Syndrome and toxic epidermal necrolysis are rare yet highly fatal conditions, where early recognition and timely intervention are critical for patient survival. As a tertiary referral center specializing in drug allergies, our institution aimed to contribute to literature and enhance awareness among healthcare professionals by sharing our single-center experience and treatment approaches in managing these conditions.

## 2. Materials and methods

This single-center, retrospective study included patients with a diagnosis of SJS/TEN at Ankara Bilkent City Hospital between August 2019 and January 2024. Ethics committee approval was obtained before the study began (NO:TABED 1-24-117). All authors signed the Declaration of Helsinki. Informed consent forms were obtained from the patients’ relatives before diagnostic tests were performed and for use of their clinical information.

The diagnosis of SJS/TEN was based on clinical findings, as there are no universally accepted diagnostic criteria. Patients exhibiting acute onset of mucosal involvement (at least 2 mucosal surfaces) and skin symptoms (macula, target-like, bullae or erosions, positive Nikolsky sign) with involvement of less than 10% of the total body surface area were classified as SJS, those with involvement of more than 30% as TEN and those with involvement of 10%–30% as SJS/TEN overlap[8]. Drugs that had been taken within eight weeks preceding the onset of symptoms were considered suspicious, excluding those that had been taken months or years before[5].

Patient information was obtained from the electronic database of our hospital, including age, gender, comorbidities, percentage of total body surface area involvement, mucosal involvement, treatment, duration of hospitalization, skin biopsy results, laboratory results (complete blood count, renal and liver function tests, bacteriological and viral examinations) and postdischarge sequelae. Admission complaints, the triggering drug and the time between beginning the drug and the onset of symptoms were obtained from clinical records and recorded on standard data collection forms. The SCORTEN (a severity of illness score specified for TEN) scoring system, a standard prognostic tool, was used to predict TEN-related mortality [9]. Patients with missing file information or those with alternative diagnoses were excluded from the study.

### 2.1. Diagnostic tests

If the clinical condition of the patient was appropriate, a skin biopsy was obtained after receiving informed consent from the patient. One skin biopsy was taken from the side of the vesicle and sent for routine histopathology, while another was taken from the perivesicular lesion area and sent for immunofluorescence examination before treatment.

### 2.2. Investigation of etiology

To identify responsible drugs, the history of drug use, the time interval between drug use and symptoms and the clinical course after discontinuation of the suspected drug were evaluated. Whenever possible, patch testing with suspected drugs was performed after obtaining parental consent. Patch tests were carried out at least six weeks after the resolution of symptoms. In accordance with the European Network for Drug Allergy guidelines, the patch tests were prepared homogeneously with petrolatum (solid Vaseline) at the suggested concentration, and each drug was placed in an aluminum chamber (Finn chamber) and adhered to the patient’s back. Petrolatum was used as a negative control. Two measurements were performed for each patient, the first after removal of the patch test on the second day and the second at hour 72 for reevaluation [10]. Serologic tests for Epstein–Barr virus, Cytomegalovirus, Herpes Simplex virus types 1 and 2, Varicella Zoster virus, Parvovirus, Rubella, Hepatitis A and B and Mycoplasma were performed to identify the responsible infectious agent in patients with symptoms of a recent infection. If patients had both infectious symptoms and positive serologic test results and there was no history of drug use, the etiology was attributed to infection.

## 3. Results

### 3.1. Patient characteristics

Twelve patients met the inclusion criteria. The patients’ demographic characteristics, responsible agents, time between the responsible drug and the onset of symptoms, comorbid diseases and mucosal involvement are presented in Table 1. Eight of the patients were female (8/12), and the median age at presentation was 10.5 years (IQR:6–16). Five patients were diagnosed with SJS, four with SJS/TEN overlap and three with TEN.

### 3.2. Causes

Ten of the 12 patients had a history of drug use. One patient was serologically positive for Mycoplasma pneumonia and one was positive for rhinovirus. Group A beta hemolytic streptococcus (GABS) was isolated in the throat culture of one patient; 6 patients (50%) had a history of beta lactam group antibiotic use and 2 (16.6 %) had a history of non-beta lactam group antibiotic use (Table 1).

Two patients were on allopurinol, two were on proton pump inhibitors (PPI), one was on valproic acid (VPA) and one was on lamotrigine. The interval between the first administration of the responsible drug and symptom onset was between 4 days and 6 weeks. The responsible drugs and the time between use and the onset of symptoms are listed in Table 1.

### 3.3. Clinical presentation

**Figure 1:**
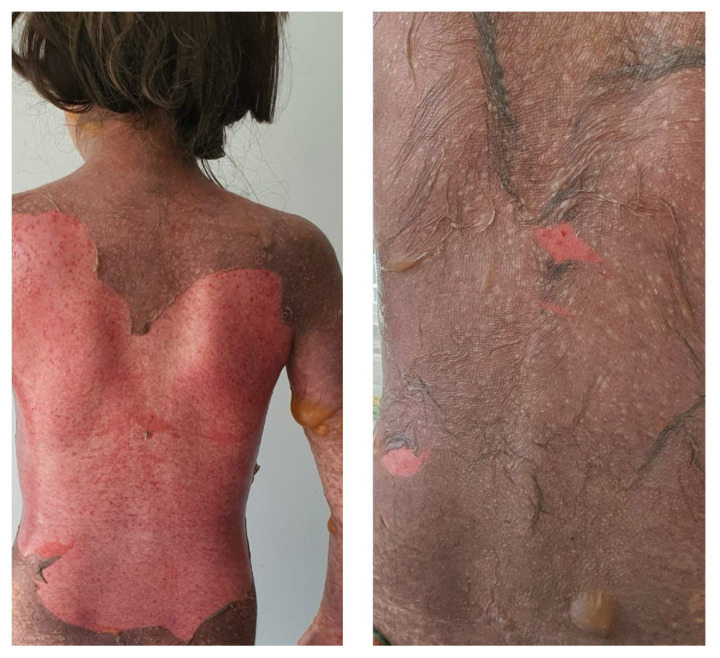
Epidermal detachment and bullous rash in a case with TEN.

**Figure 2:**
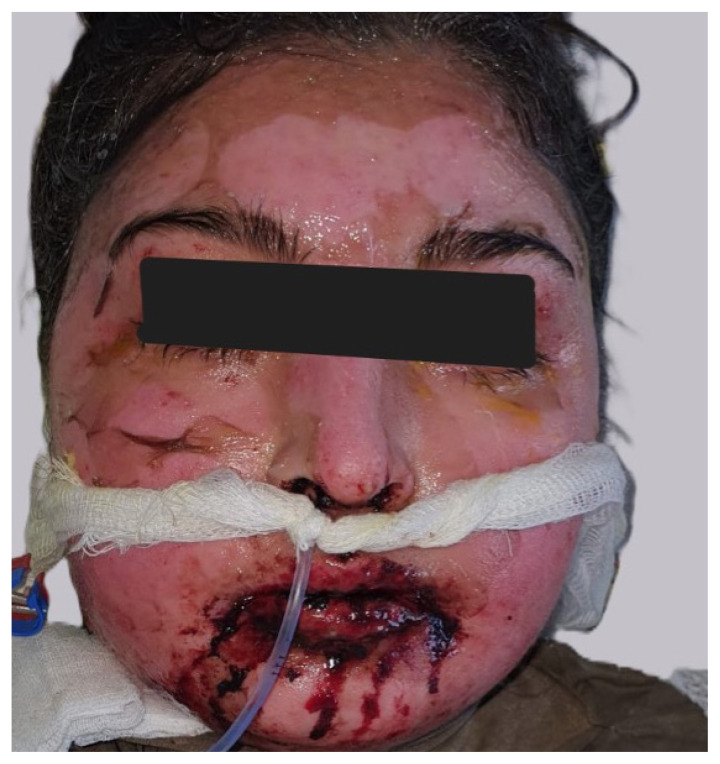
Oral mucosal lesions in a patient with TEN.

All patients had both mucosal and skin involvement. All patients had maculopapular exanthema, bullae, blisters and eroded areas, and four patients had atypical target-like lesions (Figure 1). The most common rash location was the head and neck area. All patients had oral mucosa involvement (Figure 2) and at least two involved mucosa. Nine had genital mucosa involvement, seven had anal mucosa involvement and four had ocular mucosa involvement (Table 1).

### 3.4. Diagnostic tests

Skin biopsy was performed on nine patients who consented to diagnostic tests. Biopsy findings included dermoepidermal detachment, vacuolization of the epidermal layer, subepidermal separation, keratinocyte necrosis, papillary perivascular mononuclear inflammatory infiltration and interphase dermatitis. Patch testing was performed in one patient, and the patch test with allopurinol was positive in this patient (10% concentration) (patient 5).

### 3.5. Treatment and outcome

All patients received standard in-patient treatment, including sterile wound care, intravenous fluid replacement, analgesics as well as nutritional support and were monitored in the burn unit. The patients’ SCORTENs, treatments and hospitalization durations are listed in Table 2. All 12 patients received IVIG and systemic steroids. IVIG was given at 2 g/kg divided into 2–4 days. The systemic steroid methylprednisolone was given at 1–2 mg/kg for a minimum of five days. Patients receiving steroids for more than 10 days were tapered and discontinued. Cyclosporine (CSA) was given to three TEN and four SJS/TEN overlap patients at a dose of 3–5 mg/kg/day for 5–16 days. One patient was diagnosed with autoimmune hepatitis, and continuation of cyclosporine was deemed appropriate by gastroenterology.

Two TEN patients underwent plasmapheresis. One of these patients was a 17-year-old girl with 80% skin detachment (Patient 1), while the other was a 13-year-old girl with extensive peeling and infected lesions complicated with pseudomonas (Patient 11). Both patients were treated with IVIG, steroids and CSA as well as plasmapheresis.

All of the cases were handled by an ophthalmologist within the first day of admission. Each patient was administered topical moisturizer, topical corticosteroid drops (loteprednol, dexamethasone) to reduce conjunctival inflammation, and topical broad-spectrum prophylactic antibiotics (moxifloxacin, netilmicin) in case of corneal damage and risk of infection.

Moisturizer was applied to the entire epidermis and topical antimicrobial medication was applied to the dead tissue. Nonadhesive protective layers (wet gauze dressing) were applied to SJS/TEN overlap and TEN patients. Local care was applied to the mucosa with moisturizer and mupirocin. An antimicrobial contact dressing containing 15% elemental silver was administered to the patient with TEN who presented with 80% total body surface area involvement (Patient 1). Wound care was performed using cleansing solutions comprising polyhexanide and poloxamer.

One of the patients died due to sepsis and multiorgan failure (Patient 11). A 13-year-old girl, who was followed up in an external center for eight days, presented with infected skin lesions colonized with pseudomonas.

The most common long-term sequelae were dermatologic. Two patients had persistent skin dyspigmentation (Patients 1 and 5), and two had onycholysis (Patients 1 and 10).

Amniotic membrane transplantation was performed in one case with an epithelial defect and corneal ulcer (Patient 12). There were no visual sequelae, and this patient is being followed up.

## 4. Discussion

This study involved 12 SJS/TEN patients followed in a single center. Five were evaluated as SJS, four as SJS/TEN overlap and three as TEN. While SJS/TEN is rare in children, it can lead to morbidity and mortality and must be treated early and appropriately.

Previous studies have reported a higher incidence of SJS than TEN, but in our study, more than half of the patients were diagnosed with SJS/TEN overlap and TEN [11,12]. This may be due to the fact that our hospital is a tertiary care center with a burn unit, and complicated patients are referred to our center. Thus, our study contributes to the literature by including complicated pediatric TEN and SJS/TEN overlap cases.

The pathogenesis of TEN/SJS is not fully understood. It is considered a T cell-mediated, type IV hypersensitivity response. T cells are activated by binding drugs to T cell receptors presented by antigen-presenting cells. Currently, three major hypotheses explain the mechanisms of T cell activation: the hapten/pro-hapten model, the pharmacological interaction (p-i) concept, and the modified peptide model. Most drugs and their metabolites function as pro-haptens and do not act independently as haptens. Responsible drug or drug metabolite interacts with HLA proteins on keratinocytes and CD8+ T cells in the epidermis are activated. The activated CD8+ T cells produce cytolytic peptides such as granulysin, which cause keratinocyte death. In this process, cytotoxic proteins such as perforin/granzyme are also released[13]. Genetic risk factors that may be drug-specific in SJS/TEN have been reported with certain HLA alleles. Examples include the relationship between HLA B58:01 and allopurinol, HLA B15:02 and carbamazepine, HLA B15:02 and phenytoin, HLA B57:01 and abacavir[14].

Drugs have been shown to be the most important factor in the pathogenesis of SJS/TEN. In the EuroSCAR study, one of the largest multicenter international case-control studies ever published, sulfonamides and anticonvulsants (phenobarbital, lamotrigine, and carbamazepine) were reported as the most frequently responsible drugs [4]. In a 20-year pediatric case series, antiepileptics (60%; most commonly carbamazepine, followed by phenytoin, phenobarbital, and levetiracetam) and antibiotics (26.6%) were found to contribute to the etiology of SJS/TEN [11]. Consistent with the literature, 10 of our patients had a history of drug use (83.3%). Antibiotics, especially beta lactams, were the most common drugs used by the patients in our study. In a multicenter pediatric study conducted in our country, antibiotics were found to be the most common agent (47.5%)[15]. Our patients have been observed to receive antibiotics secondary to infection. In cases of immune activation, stimuli such as infection may promote the expansion of memory T cells active against self-antigens. However, in many of these cases, it may be difficult to determine whether SJS and TEN occur in response to infection or to treatment. Studies reporting absence of representative symptoms in animal models deficient in CD8+ T cells reinforce the central role of cytotoxic T cells but do not explain the mechanism of their activation. Infectious agents have also been shown to increase Fas ligand expression or susceptibility to Fas ligand-mediated apoptosis[16].

Two patients were on allopurinol, and one of these patients showed patch test positivity. This patient developed SJS/TEN overlap and then SJS two years later due to allopurinol. A study of 92 SJS/TEN patients published in 2022 reported similar results, as allopurinol was the most common agent leading to SJS/TEN after antibiotics and antiepileptics (20/92, 21.7%)[17]. Two of the patients had antiepileptic (VPA and lamotrigine) use. In a 16-patient pediatric cohort in 2016, lamotrigine was shown to be the responsible agent in three patients and VPA in one [18]. In a multicenter study from Turkey, 34.3% of patients had antiepileptic use [15]. This is unsurprising, as antiepileptics (especially the aromatic group) have been shown to be associated with severe cutaneous drug reactions, and thus caution should be exercised when using these drugs with patients.

PPI (pantoprazole, lansoprazole) use was present in two patients followed up with TEN. Cases of SJS/TEN associated with omeprazole and lansoprazole use have been reported in the literature [19,20].

Two of our patients had no history of drug use. One was followed up with TEN, and rhinovirus was isolated in the respiratory viral panel. The other patient had SJS, and GABS was isolated in a throat culture. Although drugs are a more common factor in the pathogenesis of SJS/TEN, infectious agents are also encountered. Koh et al. conducted a study on 15 pediatric patients with SJS/TEN, finding Mycoplasma positivity in three(12). In other studies, GABS and other bacterial-viral agents have been shown as etiologic agents in SJS/TEN [5,15,21].

In SJS/TEN, the time between drug intake and the onset of clinical symptoms has been reported to be mainly between 4–24 days, with a maximum period of eight weeks [5,22]. In our study, this period was 4–42 days. Consistent with the literature, the duration was shorter for antibiotics (3–16 days) and longer for other drugs (antiepileptics and PPIs) [23].

Mortality is lower in children compared to adults. The mortality rate for TEN ranges between 25 and 35% [24,25]. In our study, one patient who was followed up with TEN died due to sepsis (SCORTEN of 4).

Ocular sequelae have been frequently reported and may be severe enough to progress to lagophthalmos and blindness [26]. We observed ocular involvement in four patients. One patient with a corneal epithelial defect recovered with supportive treatment, while another underwent amniotic membrane transplantation. None of our patients developed visual sequelae in the long term. These findings highlight the importance of early ophthalmological consultation in patients with SJS/TEN because of the potential for serious ocular complications.

Cutaneous sequelae are also common. There is a high incidence of dyspigmentation (14%–100%)—hypopigmentation, hyperpigmentation, or both—with an even higher incidence in the pediatric age group[27]. In our study, persistent dyspigmentation developed in two TEN patients (16.6%). Involvement of the nail bed may lead to nail loss with onycholysis or onychodystrophy. We observed nail dystrophy as a long-term sequela in two patients (16.6 %). To prevent nosocomial infections, skin care and ensuring the barrier function of the skin in the acute period are crucial. Long-term dermatological follow-up is important to manage long-term sequelae.

The multisystem involvement and high morbidity and mortality of TEN/SJS necessitate high-dependency, multidisciplinary care of these patients in a specialized burns unit or intensive care unit. The most important aspect of the management of TEN/SJS remains; early diagnosis, identification and withdrawal of culprit medication, supportive care, and specialist wound and multidisciplinary care. Although the role of systemic corticosteroids in the treatment of TEN/SJS is controversial, they have been shown to be useful for preventing inflammation in the early stages of the disease and to reduce some biomarkers of inflammation (IFN-IL-6) in TEN/SJS[28].To benefit from this effect, we gave steroids to all our patients. There are promising studies showing that IVIG can effectively block the interaction between the Fas receptor and Fas ligand, a reported pathway of keratinocyte death. In a systematic review conducted in children, no mortality was found in 33 patients who received IVIG treatment given at 0.25–1.5 g/kg/day for 1–5 days [29]. In addition, other retrospective studies have found that IVIG use in children is associated with shorter hospital stays and faster wound healing[30]. Therefore, we opted to administer IVIG to all our patients during the early phase of treatment. A meta-analysis published in 2016 showed that the combination of IVIG and steroids had a positive effect on survival and recovery times in SJS/TEN patients [31]. In another meta-analysis, the combination of IVIG and steroids was shown to yield better results than supportive treatment alone [32]. However, these are mostly adult data, and there have been no large-scale pediatric studies. In our study, all 12 patients received steroid and IVIG treatment. There is no consensus on this issue yet, requiring more research, particularly on pediatric patients.

In our study, all three patients with TEN and four patients with SJS/TEN overlap received CSA in addition to steroids and IVIG. CSA inhibits the activation of CD4+ T cells and CD8+ T cells in the early stages, inhibiting the release of granulosin, granzyme B and perforin; prevents keratinocyte death and apoptosis by suppressing T cell-mediated immunity . In a meta-analysis conducted in 2017, none of the 40 patients who received cyclosporine; a significant decrease in mortality was observed compared to supportive care[33]. Cyclosporine treatment is recommended to be preferred in the early period due to progressive epidermal necrolysis. We preferred it in our TEN and SJS/TEN overlap patients to prevent the progression of epidermal necrolysis. In a series of 44 adult SJS/TEN patients published in 2017, CSA was found to reduce mortality [34]. Although there are no large-scale pediatric studies on CSA, it has been shown to accelerate epithelialization and reduce mortality in case series. We observed no side effects in our patients due to the low dose and short duration of use. While there have been few studies on CSA in pediatric patients, it offers promise, especially in cases of TEN, due to its accelerating effect on clinical improvement and epithelialization and favorable side effect profile.

Plasmapheresis has been evaluated as a treatment option in case reports and series including a small number of SJS/TEN patients. Plasmapheresis can release not only circulating drugs but also their metabolites or inflammatory mediators such as cytokines and is one of the adjuvant treatment methods in TEN. The use of plasma exchange is a relatively safe intervention in more severe forms of TEN due to the presence of inflammatory cytokines, autoantibodies, expressed immune complexes, or other toxic substances that cannot be removed by dialysis. According to epidemiological studies on TEN conducted in Japan between 2005 and 2007, plasmapheresis is used only as a last resort in TEN patients who do not improve with standard treatment, including high-dose corticosteroids or adequate IVIG, and in TEN patients with more than 70% BSA involvement [35]. In a 2017 study on 12 TEN patients (2 of whom were pediatric), the combination of IVIG and CSA was found to be safe and effective [36].Plasmapheresis was applied to our two patients with 70–80% BSA involvement who showed insufficient clinical response to IVIG, steroids, and CSA. Further studies are needed to determine its effectiveness.

In this study, we present severe pediatric cases, mostly TEN and SJS/TEN overlap, from a single center, following a multidisciplinary approach in a burn unit. The strength of our study is the inclusion of a single-center pediatric series treated with CSA and plasmapheresis. The weakness of the study is that it is retrospective. However, we were able to minimize this limitation because the patients were followed and treated according to standard protocols, and their information was recorded in standard data forms.

SJS/TEN are extremely rare but life-threatening cutaneous reactions. Although most commonly caused by antibiotic use, infectious agents may also be responsible for their etiology. It is crucial to practice rational drug use, avoiding unnecessary drug prescribing. Patients on high-risk drugs for SJS/TEN should be monitored for potential side effects. Discontinuation of the responsible drug and supportive treatment are the primary treatment strategies. SJS/TEN treatment involves a multidisciplinary approach and, if necessary, follow-up in the burn unit. Early initiation of CSA in TEN patients is associated with positive outcomes. However, further studies are needed to evaluate the efficacy of other treatment options, such as IVIG, steroids, plasmapheresis, and CSA.

## Figures and Tables

**Table 1:** Demographic and clinical characteristics of patients.

Patients No	Age (Year) Sex	Diagnosis (body percentage involved)	Culprit Drug/Cause	Indication for culprit drugs	Mucosal Involvement	Onset(Days After Starting Drug)	Comorbid Disease
**1**	17, F	TEN (%80)	Macrolid Lansoprazole	Upper respiratory tract infection Gastritis	Genital Anal Oral	14 days	Henoch Schloein Purpura
**2**	6, M	SJS	Allopurinol	Hyperuricemia	Oral Anal Oculer	4 weeks	Chronic kidney disease
**3**	5,F	SJS	Sulbactam ampicillin	Upper respiratory tract infection	Oral Genital	10 days	Acute liver failure
**4**	11,M	SJS	Amoxicillin clavulonate	Upper respiratory tract infection	Oral Genital anal	7 days	
**5**	13,F	SJS/TEN overlap	Allopurinol	Hyperuricemia	Oral Genital Anal Oculer	4 weeks	Alagille syndrome
**6**	6,F	SJS	Group A streptococci		Oral Genital		
**7**	13,F	SJS	Valproic acid Amoxicillin clavulonate Mycoplasma pneumonia	Epilepsy Upper respiratory tract infection	Oral Genital	Valproic acid, 6 weeks Amoxicillin clavulonate, 6 days	
**8**	9,M	SJS/TEN overlap	Lamotrigine	Epilepsy	Oral Genital Oculer	15 days	
**9**	16,F	TEN(%40)	Meropenem Vancomycine	Meningitis	Anal Oculer Oral	Meropenem, 6 days Vancomycine, 13 days	Fallot tetralogy Meningitis
**10**	16,M	SJS/TEN overlap	Pantoprazole Amoxicillin clavulonate	Gastritis Upper respiratory tract infection	Oral Genital	Pantoprazole, 4 weeks Amoxicillin clavulonate, 4 days	Autoimmune hepatitis
**11**	13,F	TEN(%70–80)	Rhinovirus		Oral Anal Genital		
**12**	10,F	SJS/TEN overlap	Amoxicillin clavulonate	Upper respiratory tract infection	Oral Genital Oculer	4 days	

M: male, F: female

**Table 2:** Treatment regimens and outcomes in patients with SJS/TEN.

Patients No	Pediatric SCORTEN	The length of day in ICU	The length of day in hospital	Steroid	IVIG	Cyclosporine	Plasmapheresis	Outcome
**1**	2	27	37	+	+	+	+	Survived
**2**		24	64	+	+	−	−	Survived
**3**		8	24	+	+	−	−	Survived
**4**		7	9	+	+	−	−	Survived
**5**	4	11	19	+	+	+	−	Survived
**6**		3	15	+	+	−	−	Survived
**7**		7	18	+	+	−	−	Survived
**8**	3	12	20	+	+	+	−	Survived
**9**	2	13	23	+	+	+	−	Survived
**10**	1	7	42	+	+	+	−	Survived
**11**	4	12	12	+	+	+	+	Died
**12**	1	12	18	+	+	+	_	Survived

IVIG: Intravenous immunoglobuline, ICU:Intensive care unit
