# Erratum to “Evaluation of clinical features and treatment modality of pediatric patients with Steven Johnson syndrome/toxic epidermal necrolysis: a single-center experience” [Turkish Journal of Medical Sciences 55 (2) 2025 461–469]

**DOI:** 10.55730/1300-0144.6031

**Published:** 2025-06-01

**Authors:** Funda AYTEKİN GÜVENİR, Vildan Selin ÇAYHAN, Selman Kürşat BALCI, Ragıp DERE, Hatice Irmak ÇELİK, Serhat EMEKSİZ, Ahmet SELMANOĞLU, Zeynep ŞENGÜL EMEKSİZ, Emrah ŞENEL, Emine DİBEK MISIRLIOĞLU

**Affiliations:** 1 Division of Pediatric Allergy and Immunology, Department of Pediatrics, Ankara Bilkent City Hospital, Ankara, Turkiye; 2 Department of Pediatric Surgery, Ankara Bilkent City Hospital, Ankara, Turkiye; 3 Division of Pediatric Intensive Care, Department of Pediatrics, Ankara Bilkent City Hospital, Ankara, Turkiye

In the original article, the ORCID of one of the co-authors, Hatice Irmak ÇELİK, was incorrectly reported by the corresponding author. At the request of the authors, the ORCID has been corrected to reflect the accurate information.

This correction does not affect the content or conclusions of the article. The authors apologize for this oversight.

